# Cardioprotective Effect of Nicorandil, a Mitochondrial ATP-Sensitive Potassium Channel Opener, Prolongs Survival in HSPB5 R120G Transgenic Mice

**DOI:** 10.1371/journal.pone.0018922

**Published:** 2011-04-25

**Authors:** Atsushi Sanbe, Tetsuro Marunouchi, Junji Yamauchi, Kouichi Tanonaka, Hideo Nishigori, Akito Tanoue

**Affiliations:** 1 Department of Pharmacotherapeutics, School of Pharmacy, Iwate Medical University, Iwate, Japan; 2 Department of Pharmacology, National Research Institute for Child Health and Development, Tokyo, Japan; 3 Department of Pharmacology, Tokyo University of Pharmacy and Life Science, Tokyo, Japan; University of Bristol, United Kingdom

## Abstract

**Background:**

Transgenic (TG) mice with overexpression of an arg120gly (R120G) missense mutation in HSPB5 display desmin-related cardiomyopathy, which is characterized by formation of aggresomes. It is also known that progressive mitochondrial abnormalities and apoptotic cell death occur in the hearts of R120G TG mice. The role of mitochondrial dysfunction and apoptosis in disease progression, however, remains uncertain.

**Methods and Results:**

Mitochondrial abnormalities and apoptotic cell death induced by overexpression of HSPB5 R120G were analyzed in neonatal rat cardiomyocytes. Overexpression of mutant HSPB5 led to development of aggresomes with a concomitant reduction in cell viability in the myocytes. Overexpression of mutant HSPB5 induced a reduction in the cytochrome c level in the mitochondrial fraction and a corresponding increase in the cytoplasmic fraction in the myocytes. Down-regulation of BCL2 and up-regulation of BAX were detected in the myocytes expressing the mutant HSPB5. Concomitant with mitochondrial abnormality, the activation of caspase-3 and increased apoptotic cell death was observed. Cell viability was dose-dependently recovered in myocytes overexpressing HSPB5 R120G by treatment with nicorandil a mitochondrial ATP-sensitive potassium channel opener. Nicorandil treatment also inhibited the increase in BAX, the decrease in BCL2, activation of caspase-3 and apoptotic cell death by mutant HSPB5. To confirm the results of the *in-vitro* study, we analyzed the effect of nicorandil in HSPB5 R120G TG mice. Nicorandil treatment appeared to reduce mitochondrial impairment and apoptotic cell death and prolonged survival in HSPB5 R120G TG mice.

**Conclusions:**

Nicorandil may prolong survival in HSPB5 R120G TG mice by protecting against mitochondrial impairments.

## Introduction

The anti-anginal agent nicorandil is known as an opener of the ATP-sensitive potassium (KATP) channel with a nitrate moiety [1]. A significant amount of clinical evidence has demonstrated that nicorandil protects the heart against ischemic injury [1], improves the recovery of post-ischemic contractile dysfunction and can reduce infarct size in several animal models [2]. The impact of nicorandil in angina (IONA), a randomized and placebo-controlled study, has shown that nicorandil reduced the incidence of major cardiovascular events in patients with angina pectoris [1], [3]. Although it remains uncertain whether nicorandil has an infarct-limiting effect in humans, a great deal of attention has been attracted to molecular mechanisms by which nicorandil exerts a cardioprotective action. Besides the beneficial hemodynamic effects, such as increased coronary blood flow and reduced vascular resistance, recent studies suggest that the cardioprotective effects of nicorandil are mediated by activation of mitochondrial ATP-sensitive potassium (mitoK(ATP)) channels in myocytes [2], [4]. Many studies suggest that the mitoK(ATP) channels are implicated in the mechanism(s) of ischemic preconditioning (IPC), apoptosis and mitochondrial matrix swelling [5], while it's physiological role, functional regulation and exact structure remain to be determined [5].

The R120G missense mutation in heat shock protein (HSP) B5 (also known as alpha-B-crystallin) can cause desmin-related cardiomyopathy (DRM) [6]. This disease, which is characterized by the formation of aggregates containing HSPB5 and desmin, is a misfolded protein-related disease that can be recapitulated in transgenic (TG) mice by expressing the mutant HSPB5 R120G (R120G) protein specifically in the cardiomyocytes [7]. We showed that R120G caused perinuclear aggresome formation and that these aggresomes contained pre-amyloid oligomer intermediates (amyloid oligomers) [8]. These results suggest that DRM caused by the HSPB5 mutation is a subclass of aggresomal and amyloid-related diseases [8]. Our previous study also suggests that cellular toxicity induced by amyloid oligomers is associated with mitochondrial function as well as induction of apoptotic cell death by cytochrome c release from mitochondria [8], [9]. Pharmacological intervention such as grenylgrenylaceton (GGA), which can enhance HSP(s) induction and can attenuate amyloid oligomer formation, showed an effectiveness towards cardiac disease, such as reduced cardiac function, cardiac hypertrophy, interstitial fibrosis, and what appeared to be apoptotic cell death in mutant R120G TG mice [8]. It is uncertain, however, whether inhibition of the apoptotic cell death is sufficient for treatment of DRM.

In this study, we showed that nicorandil treatment could inhibit development of mitochondrial abnormalities as well as apoptotic cell death via a mitoK(ATP) channel opening in this model although levels of protein aggregates and amyloid oligomers were unchanged. Concomitant inhibition of apoptotic cell death and blockade of alteration levels of BCL family members such as BCL2 and BAX were observed in the nicorandil-treated R120G TG mice. These results imply that mitochondria protection as well as inhibition of subsequent apoptotic cell death by nicorandil, a mitoK(ATP) channel opener, may represent a new therapeutic strategy for treating patients with DRM.

## Materials and Methods

### Recombinant protein

To produce a recombinant protein, His epitope-tagged wild-type HSPB5-FLAG, HSPB5 R120G-FLAG were overexpressed in BL21 cells (Invitrogen, Carlsbad, CA, USA) using the pET system (Novagen, Madison, WI) and purified with a Ni-NTA column (Qiagen, Santa Clarita, CA) as described previously [10]. The amyloid oligomer level, each recombinant protein or protein mixture with nicorandil was incubated and blotted on a nitrocellulose membrane and quantified as described previously [10].

### Cardiomyocyte cultures and adenovirus infection

Rat neonatal cardiomyocytes were isolated using the Worthington Cardiomyocyte Isolation System (Worthington Biochemical Corporation, Lakewood, NJ). After isolation of rat neonatal cardiomyocytes, cells were grown on glass slides coated with a gelatin, as described previously [8]. The cells were normally infected at a multiplicity of infection of 10 for each virus except where indicated. Replication-deficient recombinant adenoviruses were made using an AdEasy system (Agilent Technologies, Palo Alto, CA), as described previously [10], [11].

### Immunohistochemistry

Immunohistochemical analyses were performed as described previously [8]. Alexa488-conjugated anti-rabbit, and Alexa568-conjugated anti-mouse antibodies were purchased from Molecular Probes (Eugene, OR), Anti-HSPB5 antibody (SPA-223) from Assay Designs, Inc. (Ann Arbor, MI), and anti-cTnI antibody (MAB1691) from Millipore (Billerica, MA). The anti-oligomer antibody (A-11) was generated and used as described previously [8]. The cellular viability was measured using a 3-(4,5-dimethylthiazol-2-yl)-2,5-diphenyl tetrazolium bromide (MTT) assay [8]. Image J 1.38× was used to quantify the immunofluorescent intensity. The results from 30–50 cells were averaged for cohort comparison. The area stained with the oligomer antibody was defined, and the average pixel intensity of the cardiomyocyte was determined for comparison [8].

### Isolation of mitochondrial and cytosolic fractions

Isolation of mitochondrial and cytosolic fractions was performed as described previously [9]. Hearts were homogenized in buffer containing 250 mM sucrose, 10 mM Tris-HCl (pH 7.4), 1 mM EDTA, 1 mM Na3VO4, and Complete Protease Inhibitor Cocktail Tablets (Roche, Basel, Switzerland). The homogenate was centrifuged at 1,000×g for 10 minutes at 4°C to remove the nuclei. The supernatant was centrifuged again at 13,000×g for 30 minutes at 4°C. The pellet was washed extensively in the same buffer, centrifuged as above, and the pellet (mitochondrial fraction) was re-suspended in lysis buffer containing 150 mM NaCl, 50 mM Tris-HCl (pH 7.4), 1 mM EDTA, 1 mM Na3VO4, Complete Protease Inhibitor Cocktail Tablets (Roche, Basel, Switzerland), and 1% NP-40. Supernatant was further purified at 100,000 g for 30 min (4°C) and used as the cytosolic fraction.

### Miscellaneous methods

Sample preparation for Western blotting, gel preparation, and electrophoretic conditions have been described previously [8]. Western blot analyses were performed with the use of anti-GAPDH antibody (Chemicon International, Temecula, CA), anti-HSPB5 antibody (SPA-223, Assay Designs Inc., Ann Arbor, MI), anti-cytochrome c antibody (BD Bioscience, CA), anti-voltage dependent anion channel (VDAC) antibody (EMD Chemicals Inc., Gibbstown, NJ), and anti-BCL2 antibody (SC7382, Santa Cruz Biotechnology, Inc., Santa Cruz, CA) and anti-BAX antibody (SC6236, Santa Cruz Biotechnology, Inc.). The band intensity in the immunoblot was semi-quantified using Image J1. The filter assay for the detection of the aggregates was performed as described previously [8]. The aggregates were detected with an anti-HSPB5 antibody. TUNEL staining was carried out using an *in-situ* cell death detection kit, TMR Red (Roche, Basel, Switzerland), following the manufacturer's instructions as described previously [8]. To detect anti-annexin V positive cells as a marker of apoptotic cell death, an ApoAlert Annexin V-FITC Apoptosis Kit was used (Takara Bio Company, Shiga, Japan), following the manufacturer's instructions. The number of DAPI-labeled nuclei was counted and compared to the number of TUNEL-positive and annexin V-positive cells [9]. Echocardiography [8] and trichrome staining [8] were performed as described previously.

### Transgenic mice

Female mice with cardiac-specific overexpression of mutant HSPB5 containing the R120G mutation, driven by the α-myosin heavy chain promoter, have been described previously [8]. The TG mice were identified by PCR analysis of genomic DNA isolated from tail tips. The R120G TG mice used for all experiments were backcrossed with a C57BL/6 SLC mouse more than 10 times, and maintained on a C57BL/6 SLC background [12] (SLC Inc., Shizuoka, Japan). Non-transgenic (NTG) littermates were always used as controls for comparison. Animals were housed in microisolator cages in a pathogen-free barrier facility. All experimentation was performed under approved institutional guidelines.

### Experimental design and drug treatment

Nicorandil was kindly provided by Chugai Pharmaceutical Co., LTD (Tokyo, Japan). For the study with cardiomyocytes, nicorandil was dissolved in medium. After 24-hr of plating the primary isolated cardiomyocytes, they were infected with an adenoviral vector containing HSPB5 R120G as well as HSPB5. Nicorandil was dissolved into the culture medium 24-hr after the adenoviral infection. Ninety-six hours after infection, Western blotting, cellular viability assay and immunohistochemistry were performed. To analyze the underlying mechanism of nicorandil, diazoxide, a specific mitoK(ATP) channel opener, 5-hydroxydecanoic acid (5-HD), a specific inhibitor of the mitoK(ATP) channel, and sodium nitroprusside (SNP), a nitric oxide donor, were dissolved in media, and the cardiomyocytes were treated using the same protocol as for nicorandil.

For oral administration in the TG mice, nicorandil was dissolved in tap water and water consumption was measured for a week to adjust the dose to 15 mg/kg/day (81 mg/l of drinking water). The effects of nicorandil on cardiac disease were examined in R120G TG mice using two different protocols. In the first protocol, nicorandil treatment was started from 12 weeks of age, at which time cardiac disease is mild, in the TG mice for 4 weeks after genotyping TG mice with tail tips. In the second protocol, nicorandil was administered to the TG mice from 20 weeks of age, in which marked cardiac disease such as cardiac hypertrophy and interstitial fibrosis were observed. Western blotting, morphological analysis, immunohistochemistry and echocardiography were performed on the mice as described above at 16 weeks of age and 28 weeks of age after treatment.

### Statistics

Data are expressed as the mean ± standard error. Statistical analysis was performed using the unpaired Student's t-test and one-way or two-way ANOVA followed by a post hoc comparison with Fisher's PLSD using Statview version 5.0 software (Concepts, Inc., Berkeley, CA). Differences between groups were considered statistically significant at the level of p<0.05. Kaplan-Meier survival curves were analysed by a log-rank test using Prism version 5.0a software (GraphPad Software Inc., La Jolla, CA)

### Ethics

This study was approved by the Animal Care Committee of Iwate Medical University (Approval ID: 22-020). All experimental procedures were performed in accordance with the Guidelines of the Iwate Medical University Ethics Committee for Animal Treatment and the Guidelines for Proper Conduct of Animal Experiments by the Science Council of Japan.

## Results

### Effects of nicorandil on cellular toxicity induced by R120G in neonatal rat cardiomyocytes

**Figure 1:**
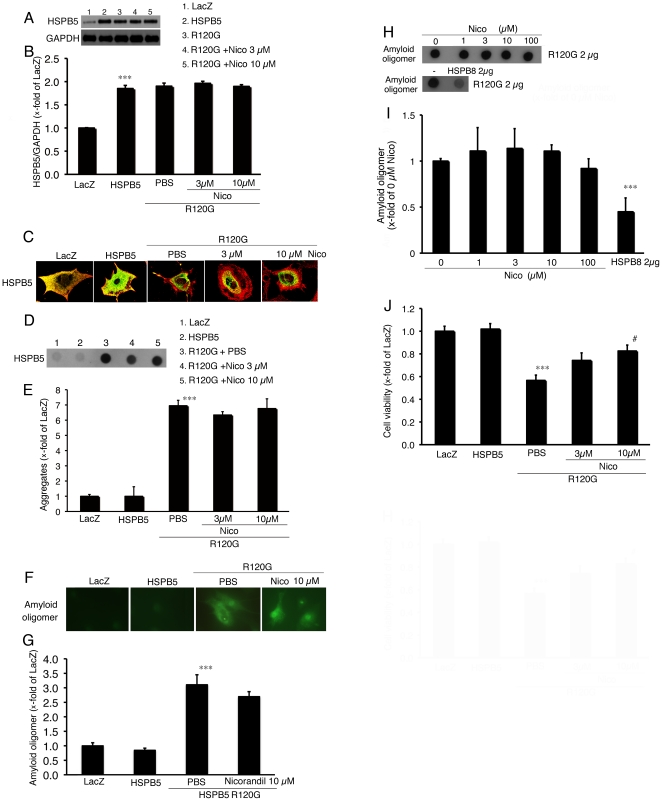
Effects of nicorandil (Nico) in neonatal rat cardiomyocytes expressing HSPB5 R120G (R120G). (A) Typical pictures of Western blot analysis. Cardiomyocytes were infected with the adenovirus vector containing the wild-type HSPB5 (HSPB5) or R120G with or without Nico treatment. (B) Quantitative analysis of HSPB5 (n = 4). Values are the x-fold increase relative to cardiomyocyte cultures infected with the adenovirus vector containing LacZ whose values are arbitrarily set to 1. (C) Representative pictures of immunohistochemical analyses of the R120G-infected cardiomyocytes. HSPB5 (green) was detected by the anti-HSPB5 antibody as described in the Materials and methods section. To distinguish between the cardiomyocytes, cardiac troponin I was stained (red). (D) Typical picture of the filter assay for the detection of the aggregates. (E) Quantitative analysis of the aggregates containing the mutant R120G protein with or without Nico treatment (n = 4). (F) Representative pictures of immunohistochemical analyses in the R120G-infected cardiomyocytes. An amyloid oligomer (green) was detected by the anti-oligomer antibody as described in the Materials and methods section. (G) Quantitative analysis of the amyloid oligomer. Amyloid oligomer levels were measured by fluorescence intensity (n = 4). (H and I) Dot blotting shows the presence of the amyloid oligomer in the recombinant R120G protein with or without Nico treatment. (J) Protective effects of Nico on the cellular viability of the R120G infected cardiomyocytes. Cellular viability was determined by the MTT assay. Values are the x-fold increase relative to the cardiomyocyte infected with LacZ whose values are arbitrarily set to 1 (n = 6). *** p<0.001 vs. the cardiomyocytes infected with LacZ, # p<0.05 vs. cardiomyocytes infected with R120G.

Effects of nicorandil on cellular toxicity induced by R120G were evaluated in neonatal rat cardiomyocytes using an adenoviral expression system. The adenovirus containing the wild-type HSPB5 gene generated twice the level of HSPB5 expression in cardiomyocytes compared to that in cardiomyocytes infected with the adenovirus-containing LacZ gene (Figure 1A and 1B). No differences of HSPB5 protein levels were seen among the cardiomyocytes infected with the wild-type HSPB5-adenovirus and the R120G-adenovirus with or without nicorandil treatment (Figure 1A and 1B). The R120G protein induced HSPB5 containing aggregates (Figure 1C–E) as well as amyloid oligomers (Figure 1E and F) in the cardiomyocytes, whereas no aggregates containing HSPB5 and amyloid oligomers were detected in the cardiomyocytes expressing the wild-type HSPB5. Levels of the aggregates and the amyloid oligomers were unchanged by treatment of nicorandil in the cardiomyocytes expressing R120G (Figure 1C–G). The amyloid oligomers may represent the primary toxic mechanism of amyloid pathogenesis, including DRM [8]. To further examine the direct effect of nicorandil on amyloid oligomer formation, we performed the dot blot analysis using recombinant R120G protein. Whereas the amyloid oligomer levels of the R120G were decreased by HSPB8, a positive control, no significant effect on amyloid oligomer levels by nicorandil treatment in dot blot analysis was observed (Figure 1H and I). While no difference in levels of the aggregates and amyloid oligomers were observed in the cardiomyocytes expressing R120G with nicorandil treatment, reduction in cell viability was prevented by nicorandil treatment in a dose-dependent manner (Figure 1J). These results suggest that nicorandil can maintain cellular viability against the challenge of R120G-induced cellular toxicity without alteration of the protein aggregate level as well as of amyloid oligomer formation.

### Release of mitochondrial cytochrome c in the cardiomyocytes infected the adenovirus vector containing the R120G

**Figure 2:**
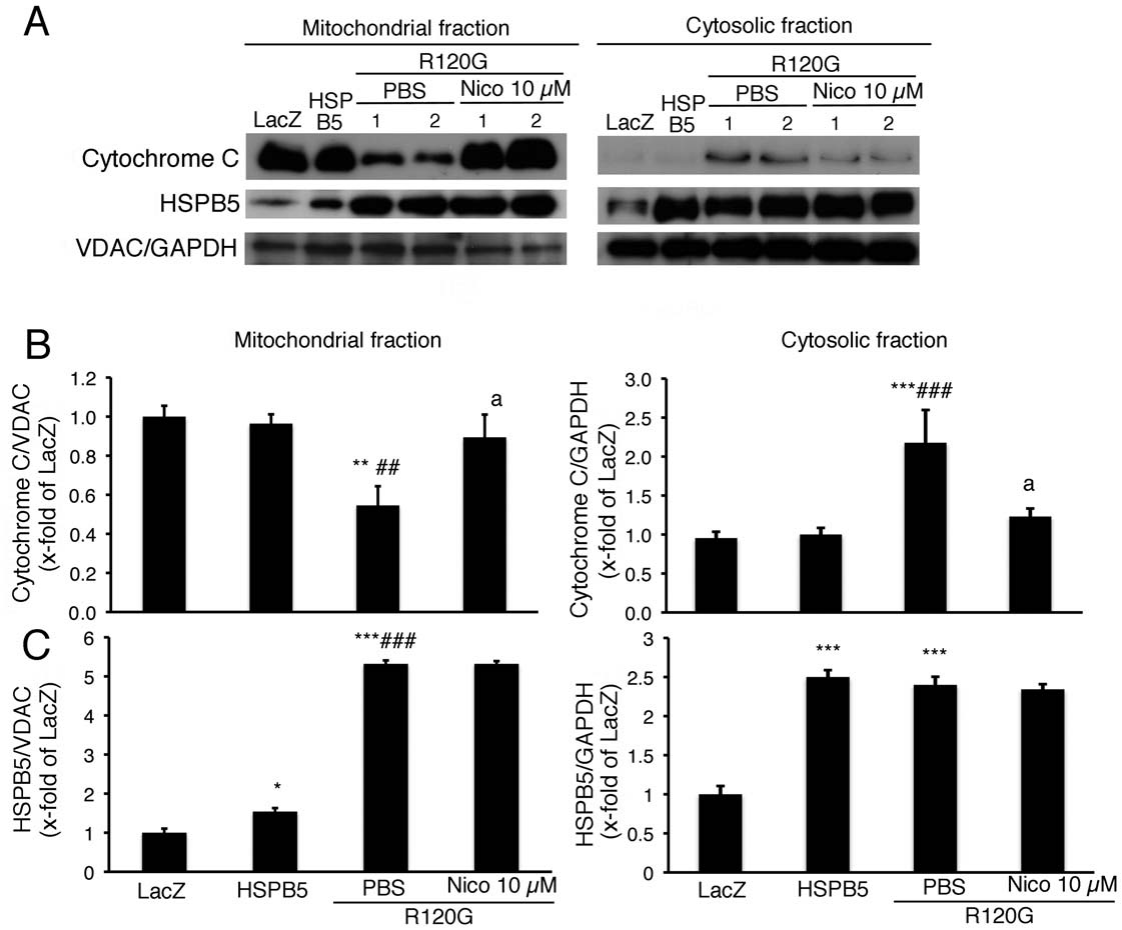
Cytochrome c levels of mitochondrial fraction isolated from the cardiomyocytes expressing HSPB5 R120G (R120G) with or without Nico treatment. (A) Typical pictures of Western blot analysis. Quantitative analysis of cytochrome c (B) and HSPB5 (C) in mitochondrial and cytosolic fractions isolated from the cardiomyocytes. GAPDH was used as a loading control in the cytosolic fraction and the voltage-dependent anion channel (VDAC) was used in mitochondrial fraction. *p<0.05, ** p<0.01, ***p<0.001 vs. the cardiomyocytes infected with LacZ (LacZ), ##p<0.01, ###p<0.001 vs. the cardiomyocytes infected with wild-type HSPB5 (HSPB5), ap<0.05 vs. cardiomyocytes infected with R120G treated with PBS. (n = 6).

Recent studies suggest that nicorandil can function as a mitoK(ATP) channel opener in the cardiomyocytes [4], which implies that nicorandil may have a protective effect on the mitochondria against cellular toxicity induced by the R120G in the cardiomyocytes. In order to evaluate the mitochondrial abnormality in the cardiomyocytes expressing R120G, the release of the cytochrome c level from mitchondria to cytosol, whose release can lead to induce nuclear apoptosis in addition to its role in oxidative phosphorylation [13], was determined in the cardiomyocytes (Figure 2A–C). Cytochrome c in the mitochondrial fraction was markedly attenuated by the overexpression of R120G while a dramatic increase in cytochrome c was observed in the cytosolic fraction (Figure 2A and B). Nicorandil treatment inhibited the release of cytochrome c from the mitochondrial fraction to cytosolic fraction (Figure 2A and B). In a previous study, we found that R120G can strongly interact with mitochondria, particularly VDAC, in the heart [9]. This result may imply that the inhibition of HSPB5 interaction with mitochondrial protein caused by nicorandil may explain the reduction in cytochrome c release from mitochondria. Mitochondrial HSPB5 protein is increased in the cardiomyocytes expressing R120G compared to that of the wild-type HSPB5 while cytosolic HSPB5 was at a similar level (Figure 2A and C). No change of HSPB5 levels was observed in either cytosolic or mitochondrial fractions between the cardiomyocytes expressing R120G with or without nicorandil treatment (Figure 2A and C). These results suggest that nicorandil can inhibit the cytochrome c release from mitochondria without alteration of HSPB5 distribution.

### Inhibition of apoptotic cell death by nicorandil treatment

**Figure 3:**
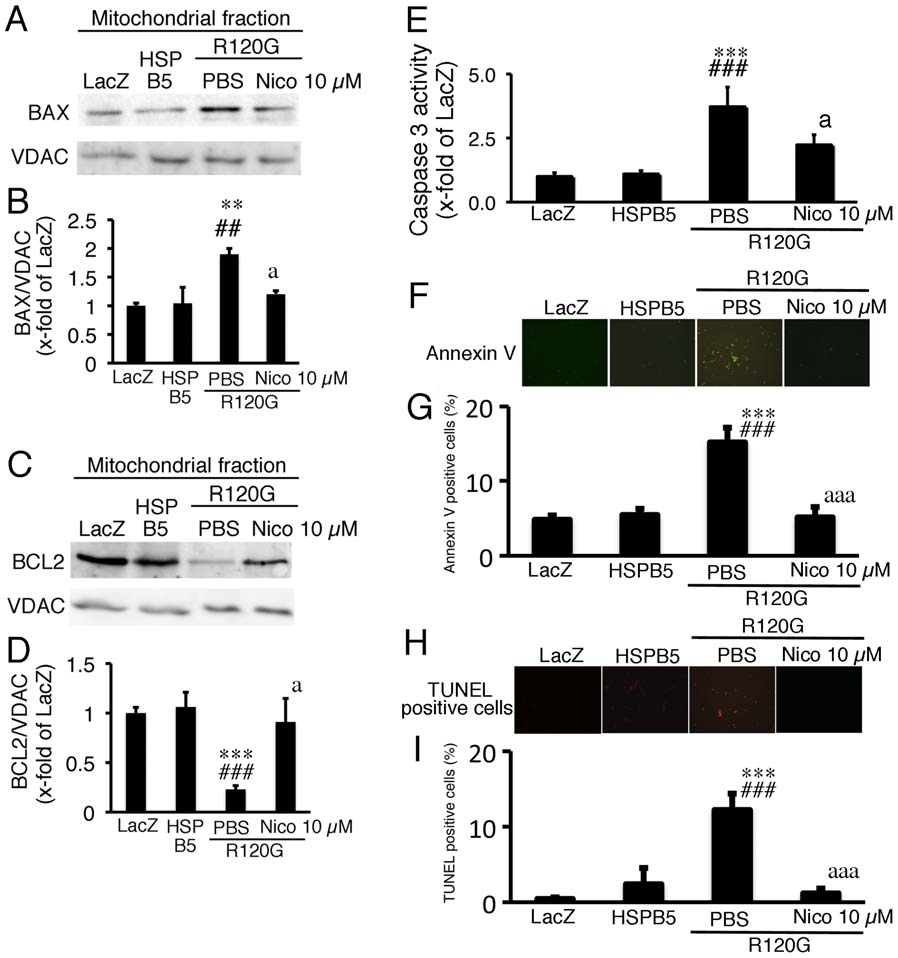
Western blot analysis of apoptosis related proteins, BAX and BCL2, and apoptotic cell death in the cardiomyocytes expressing HSPB5 R120G (R120G) with or without Nico treatment. (A) Typical pictures of Western blot analysis. (B) Quantitative analysis of BAX in mitochondrial fraction isolated from the cardiomyocytes. (C) Typical pictures of Western blot analysis. (D) Quantitative analysis of BCL2 in mitochondrial fraction isolated from the cardiomyocytes. (E) Caspase 3 activity in the cardiomyocytes. (F) Typical pictures of annexin V-positive cardiomyocytes. (G) Quantitative analysis of annexin V-positive cardiomyocytes. (H) Typical pictures of TUNEL-positive cardiomyocytes. (I) Quantitative analysis of TUNEL-positive cardiomyocytes. ** p<0.01, ***p<0.001 vs. the cardiomyocytes infected with LacZ (LacZ), ##p<0.01, ###p<0.001 vs. the cardiomyocytes infected with wild-type HSPB5 (HSPB5), ap<0.05, aaap<0.001 vs. cardiomyocytes infected with R120G treated with PBS. (n = 6).

It is known that the release of cytochrome c as well as subsequent apoptotic cell death is regulated by BCL2 family members such as BAX, an apoptosis-inducing factor, and BCL2, an anti-apoptotic factor [14]. The BCL family can form a complex between BCL2 family members and can regulate permeabilization of the mitochondrial outer membrane, which results in the release of cytochrome c and activation of the apoptotic cell death pathway [14]. Since nicorandil inhibited cytochrome c release from mitochondria, this effect may be associated with the occurrence of subsequent apoptotic cell death as well as the alteration of BCL family members in the cardiomyocytes expressing R120G. The expression of R120G induced an increase in the BAX/VDAC ratio and a decrease in the BCL2/VDAC ratio in the mitochondrial fraction from the cardiomyocytes, and nicorandil treatment blocked these alterations of the BCL family (Figure 3A–D). To examine whether nicorandil can change mRNA expression levels of these factors, we performed quantitative RT-PCR analysis. HSPB5 R120G increased the BAX/GAPDH ratio and the BCL2/GAPDH ratio compared with cardiomyocytes expressing the wild-type HSPB5 (BAX/GAPDH: 4.3±0.4 fold, BCL2/GAPDH: 1.4±0.2 fold; p<0.05). These changes in expression levels of BAX and BCL2 were also observed in the cardiomyocytes expressing R120G with nicorandil treatment compared with cardiomyocytes expressing the wild-type HSPB5 (BAX/GAPDH: 4.0±0.3 fold, BCL2/GAPDH: 1.8±0.2 fold; p<0.05). There were no differences of the expression levels of BAX and BCL2 between the cardiomyocytes expressing R120G with or without nicorandil treatment. Thus, these alterations of protein levels of BAX and BCL2 by nicorandil treatment did not exactly track the changes observed in mRNA expression. Concomitant with the alterations of the BAX/VDAC and the BCL2/VDAC ratios, R120G induced activation of caspase 3 (Figure 3E), and an increase in annexin V-positive cardiomyocytes (Figure 3F and G) and TUNEL-positive cardiomyocytes (Figure 3H and I). These results indicate that the expression of R120G can activate the mitochondrial apoptotic cell death pathway. Nicorandil treatment inhibited activation of caspase 3 and the increases in annexin V- and the TUNEL-positive cells in the cardiomyocytes expressing R120G (Figure 3A–I). These results suggest that the recovery of cell viability by nicorandil treatment can be mediated via inhibition of apoptotic cell death.

### Role of mitochondrial K(ATP) channel in cardiomyocytes expressing R120G

**Figure 4:**
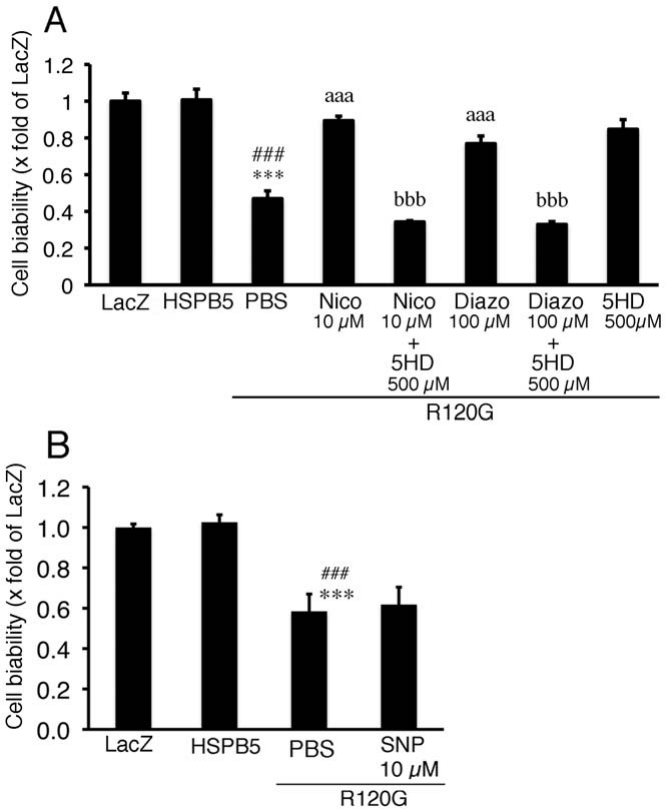
Cell viability in cardiomyocytes expressing R120G. (A) Protective effects of 10 µM Nico or 100 µM diazoxide (Diazo) on the cellular viability was inhibited by co-treatment with 500 µM 5-hydroxydecanoate (5HD). (B) No detectable effect on the cellular viability was seen by treatment with 10 µM sodium nitroprusside (SNP). Values are the x-fold increase relative to the cardiomyocyte infected with the LacZ whose values are arbitrarily set to 1. *** p<0.001, vs. the cardiomyocytes infected with LacZ (LacZ), ### p<0.001, vs. the cardiomyocytes infected with wild-type HSPB5 (HSPB5), aaa p<0.001 vs. cardiomyocytes infected with R120G treated with PBS, and bbb p<0.001 vs. cardiomyocytes infected with R120G treated with 10 µM Nico or 100 µM Diazo (n = 6).

Since it is known that nicorandil can act as a vasodilator by activating sarcolemmal and the mitoK(ATP) channel and by donating nitric oxide [15], the mechanism of the inhibitory effect of nicorandil on R120G induced cell death was analyzed in the cardiomyocytes. As described above, nicorandil prevented the reduction in cell viability induced by R120G (Figure 4A). Similar to nicorandil, diazoxide, a specific mitoK(ATP) channel opener, also inhibited the R120G-induced cell injury (Figure 4A). The protective effect of nicorandil was completely blocked by treatment with 5-HD, a specific mitoK(ATP) channel blocker (Figure 4A). In contrast to diazoxide, no protective effect was seen via treatment with SNP, a nitric oxide donor (Figure 4B). These results suggest that the mitoK(ATP) channel opening by nicorandil plays an important role in cellular protection in cardiomyocytes expressing R120G. Thus, nicorandil, the mitoK(ATP) channel opener, may protect against mitochondrial abnormalities, cellular toxicity and subsequent cell death, including apoptosis, in DRM.

### Effect of nicorandil in R120G TG mice at a relatively early stage

**Figure 5:**
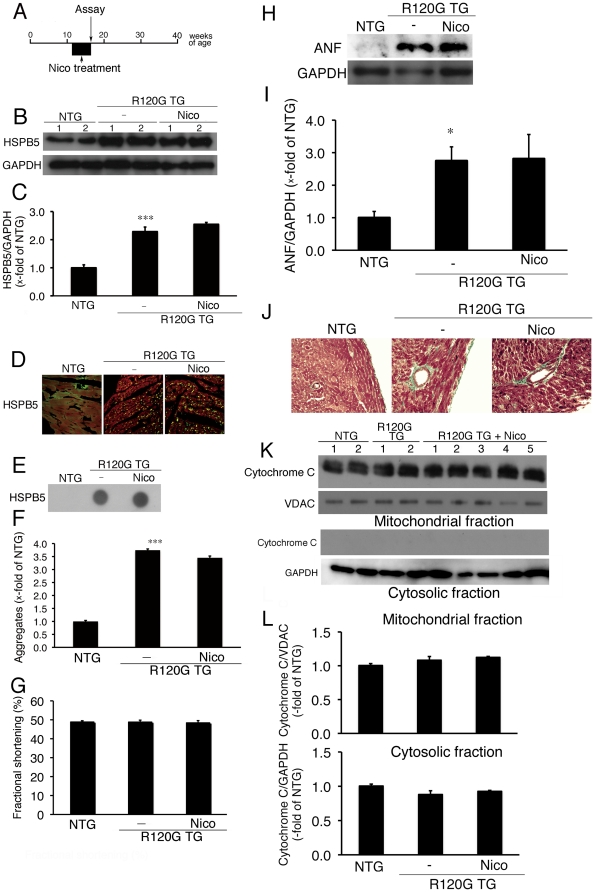
Effect of Nico (81 mg/l of drinking water) on cardiac disease at a relatively early stage in the HSPB5 R120G (R120G) TG mice. (A) Protocol was shown. Nico was administered via drinking water from 12 weeks for a total of 4 weeks in the R120G TG mice. (B) Typical pictures of Western blot analysis. (C) Quantitative analysis of HSPB5. Values are the x-fold increase relative to values in non-transgenic (NTG) mouse hearts whose values are arbitrarily set to 1. (D) Representative pictures of the immunohistochemistry are shown. (E) Typical picture of the filter assay for the detection of the aggregates containing HSPB5 proteins. (F) Quantitative analysis of the aggregates containing mutant R120G protein. (G) Fractional shortening assessed by the echocardiogram. Cardiac functional measurements were made at 16 weeks (n = 8 mice). (H) Typical pictures of Western blot analysis for ANF. (I) Quantitative analysis of ANF in the hearts in the R120G TG mice. (J) Masson's trichrome staining. (K) Cytochrome c levels of mitochondrial fraction isolated from the hearts from the R120G TG mice with or without Nico treatment. Typical pictures of Western blot analysis. Quantitative analysis of cytochrome c. GAPDH was used as a loading control in the cytosolic fraction and the voltage-dependent anion channel (VDAC) was used in the mitochondrial fraction. (L) Quantitative analysis of cytochrome c in mitochondrial and cytosolic fractions isolated from the hearts in the R120G TG mice. *p<0.05, ***p<0.001, vs. the NTG, #p<0.05 vs. the R120G TG mice (n = 6–8).

Our *in-vitro* study indicates that nicorandil can protect against mitochondrial abnormalities, cellular toxicity and subsequent cell death, including apoptosis, in DRM. To extend the results of the *in-vitro* study, we analyzed the effect of nicorandil in R120G TG mice *in vivo*. At first, nicorandil treatment was started from 12 weeks of age, in which cardiac disease including mitochondrial injury is mild, in the R120G TG mice for 4 weeks (Figure 5A). Systolic blood pressure, heart rate in a conscious state, and heart weight/body weight ratio are shown in Table 1. No differences in systolic blood pressure or heart rates were seen among NTG mice or R120G TG mice with or without nicorandil treatment (Table 1). These results imply that changes in blood pressure caused by nicorandil treatment under present experimental conditions are unlikely in the R120G TG mice. We also determined cardiac function via echocardiography in the R120G TG mice. No differences in cardiac function, such as measured fractional shortening, were observed among the NTG or R120G TG mice with or without nicorandil treatment at 16 weeks of age (Figure 5G). An increased level of HSPB5 was observed in the cardiac ventricle of the R120G TG mice compared with that in NTG mice (Figure 5B and C). No differences in the cardiac HSPB5 level between the R120G TG mice with or without nicorandil treatment were observed (Figure 5B and C). Immunohistochemical analysis showed that small aggregates containing HSPB5 were present in the R120G TG mice with or without nicorandil treatment, and aggregate level was unaffected by nicorandil treatment (Figure 5D–F). The heart weight/body weight ratio of the R120G TG mice was higher than that of NTG mice (Table 1). This increased weight ratio was unchanged by nicorandil treatment (Table 1). Similar to the heart weight/body weight ratio, ventricular ANF levels, a typical hypertrophic marker [16] (Figure 5H and I) and perivascular fibrosis (Figure 5J) were unchanged by nicorandil treatment, whereas obvious ANF induction and typical perivascular fibrosis was observed in the R120G TG mice compared to in the NTG mice. At the same time, no release of cytochrome c from the mitochondrial fraction was detected in the ventricle from the R120G TG mice (Figure 5K and L). Thus, there results imply that no effective recovery of cardiac disease by nicorandil treatment is exerted in the R120G at 16 weeks of age when the cardiac disease, including release of mitochondrial cytochrome c, is relatively mild.

**Table 1:** Heart weight and blood pressure.

	NTG	HSPB5 R120G TG	HSPB5 R120G TG+Nico
Systolic blood pressure (mmHg)	106±2	98±3	99±3
Heart rates (beats/min)	679±14	646±6	635±10
Body weight (g)	25±2	24±2	25±2
Heart weight/body weight ratio (mg/g)	4.3±0.2	5.8±0.2***	5.7±0.1

Values are mean±SEM; TG: transgenic, NTG: non-transgenic; n = 6–8 nico, nicorandil.

***p<0.001 vs. NTG)mice.

### Anti-apoptotic effect of nicorandil in HSPB5 R120G TG mice at a late stage

**Figure 6:**
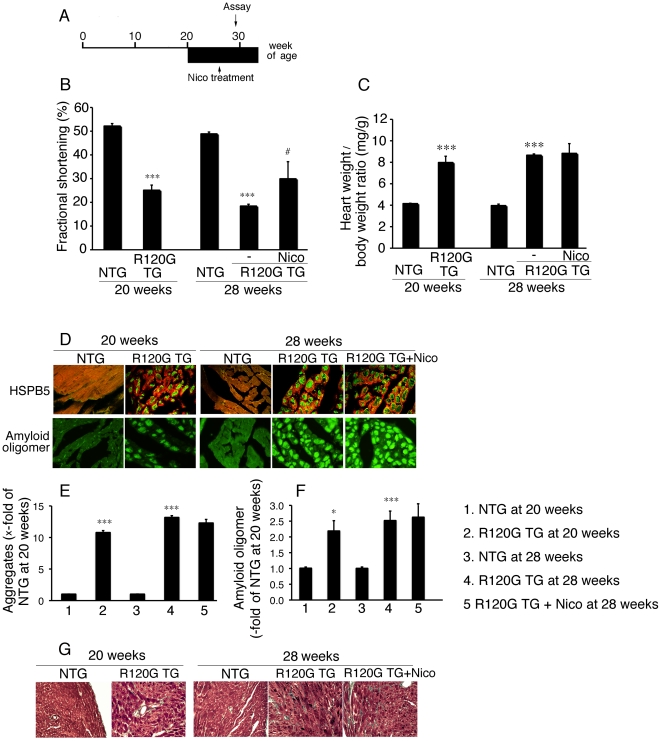
Effect of Nico (81 mg/l of drinking water) on cardiac disease at late stage in the HSPB5 R120G (R120G) TG mice. (A) Protocol was shown. Nico was administered via drinking water from 20 weeks of age in the R120G TG mice. (B) Fractional shortening assessed by the echocardiogram. Cardiac functional measurements were made at 28 weeks (n = 6–10 mice). (C) The ratios of heart weight/body weight. (n = 6–10). (D) Representative pictures of the immunohistochemistry are shown. The aggregates containing the mutant R120G protein (upper panel green) and amyloid oligomer (lower panel green) were observed in the R120G TG mice. To distinguish between the cardiomyocytes, cardiac troponin I was stained (upper panel red) (E and F) Quantitative analysis of the aggregates containing the mutant HSPB5 R120G protein (E) and amyloid oligomer (F). (n = 4). (G) Masson's trichrome stain in the R120G TG mouse heart with or without Nico treatment. * p<0.05, *** p<0.001 vs. non-transgenic (NTG) mice; # p<0.05, ### p<0.001 vs. R120G TG mice.

**Figure 7:**
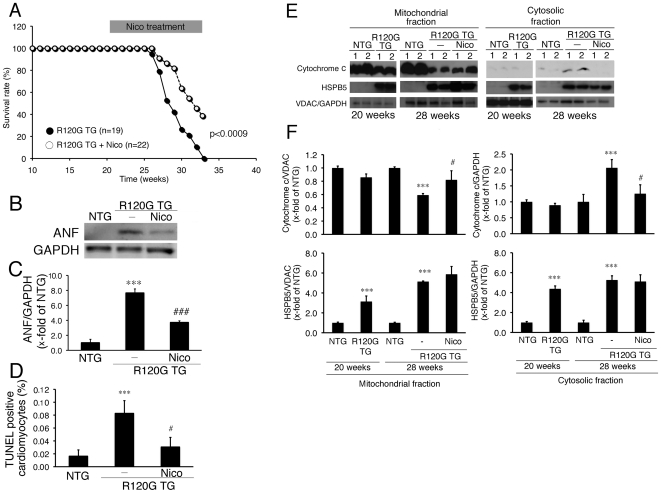
Effect of Nico (81 mg/l of drinking water) on survival rate and biochemical parameters at the late stage in the HSPB5 R120G (R120G) TG mice. (A) Survival curves. Nico effectively attenuated premature death in R120G TG mice. (B) Typical pictures of Western blot analysis for ANF. (C) Quantitative analysis of ANF in the hearts in the R120G TG mice. (D) Quantitative analysis of TUNEL-positive cardiomyocytes. (E) Typical pictures of Western blot analysis. (F) Quantitative analysis of HSPB5 and. Values are the x-fold increase relative to values in non-transgenic (NTG) mouse hearts whose values are arbitrarily set to 1. (D) Representative pictures of the immunohistochemistry are shown. The aggregates containing the mutant R120G protein are observed in R120G TG mice with or without Nico treatment. (E) Cytochrome c levels of mitochondrial fraction isolated from the R120G TG mouse heart with or without Nico treatment. (F) Typical pictures of Western blot analysis. Quantitative analysis of cytochrome c (F upper colomun) and HSPB5 (F lower column) in mitochondrial and cytosolic fraction isolated from the R120G TG mice. GAPDH was used as a loading control in the cytosolic fraction and voltage-dependent anion channel (VDAC) was used in the mitochondrial fraction. ***p<0.001 vs. non-transgenic (NTG) mice; # p<0.05, ### p<0.001 vs. R120G TG mice (survival rate: n = 19–20, and other experiments: n = 4).

**Figure 8:**
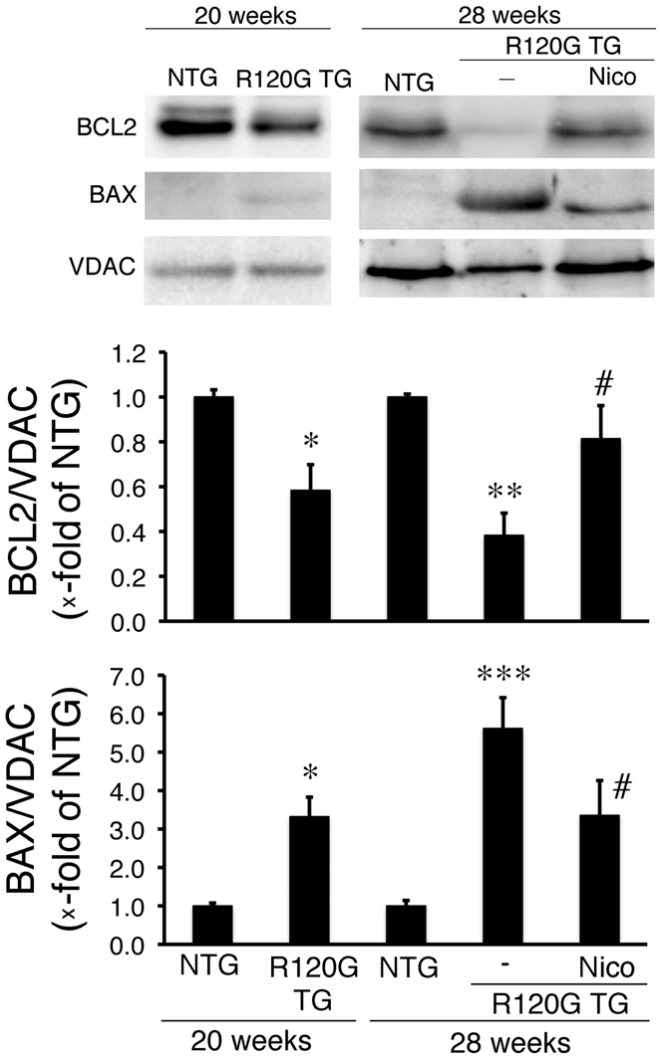
Western blot analysis of apoptosis-related proteins, BAX and BCL2, and apoptotic cell death in HSPB5 R120G (R120G) TG mice with or without Nico treatment. (A) Typical pictures of Western blot analysis. (B and C) Quantitative analysis of BAX (B) and BCL2 (C) in mitochondrial fraction isolated from the TG mouse hearts. * p<0.05, **p<0.01 vs. non-transgenic (NTG) mice; # p<0.05 vs. R120G TG mice (n = 4).

Nicorandil can inhibit the release of cytochrome c, ANF induction and concomitant cell death in the cardiomyocytes expressing the mutant HSPB5 as described above. In an *in-vivo* DRM model, no beneficial effect of nicorandil treatment was detected when cardiac disease, and mitochondrial abnormalities were relatively mild. As a next step, we examined the effects of nicorandil on DRM disease at a late stage, in which mitochondrial abnormalities have occurred (Figure 6A). Nicorandil was administered to the TG mice from 20 weeks of age (Figure 6A). At that time, cardiac disease, as manifested by reduced fractional shortening (Figure 6B), cardiac hypertrophy (Figure 6C), intracellular aggregates (Figure 6D and E), amyloid oligomers (Figure 6D and F) and interstitial fibrosis (Figure 6G) were observed (Figure 6B–G). Further progression of these pathologies were seen in the mutant TG mice at 28 weeks of age (Figure 6). Nicorandil treatment inhibited reduction in fractional shortening with no obvious effects on cardiac hypertrophy, level of the aggregates, level of the amyloid oligomers, or presence of fibrosis being detected (Figure 6B–G). However, nicorandil treatment did have beneficial effects, including, increased survival (Figure 7A), decreased ANF induction (Figure 7B and C), and decreases in TUNEL-positive cardiomyocytes (Figure 7D). Mitochondrial cytochrome c was released in the ventricle from R120G TG mice at this age (Figure 7E and F), and nicorandil treatment blocked this as well (Figure 7E and F). Concomitant with a reduction of cytochrome c being released from the mitochondria, a marked decrease in BCL2 as well as a marked increase in BAX were observed in the R120G TG mice (Figure 8A–C), and nicorandil treatment inhibited these alterations in R120G TG ventricles. These data indicate that nicorandil treatment appeared to reduce mitochondrial impairment, such as release of cytochrome c, and inhibit activation of the apoptotic pathway, such as decrease in BCL2 and increase in BAX, and inhibit the subsequent induction of apoptotic cell death, thereby prolonging the survival in R120G TG mice. Thus, nicorandil, a mitoK(ATP) channel opener, may prolong survival in R120G TG mice by protecting against mitochondrial impairments and inhibiting apoptotic cell death.

## Discussion

We have demonstrated that progressive mitochondrial abnormalities and apoptotic cell death occur in the hearts of R120G TG mice in previous studies [8], [9]. Although it is known that these abnormalities (such as mitochondrial dysfunction and apoptotic cell death) are probably associated with disease of the DRM, the role of mitochondrial dysfunction and apoptotic cell death in disease progression remains uncertain. In the present study, we analyzed the effect of nicorandil, a mitoK(ATP) channel opener, on disease progression such as mitochondrial injury and the occurrence of apoptotic cell death concomitant reduction in survival rate in the desmin-related cardiomyopathy. This must be studied carefully, because nicorandil affects the salcoremmal K(ATP) channel as well as guanylate cyclase as a NO donor in vessels [1]. As such, we employed two different experimental protocols: treatment at a relatively early stage and treatment at a relatively late stage. In the early stage, when relatively mild mitochondrial abnormalities are observed in the cardiomyocytes, no beneficial effect on cardiac hypertrophy, fibrosis, or ANF induction was detected by nicorandil treatment. In contrast, nicorandil treatment prolonged survival, reduced mitochondrial injury, and inhibited apoptotic cell death in R120G TG mice at a relatively late stage when we commenced nicorandil treatment at 20 weeks of age, when mitochondrial abnormalities are observed in the cardiomyocytes. In this experiment, we commenced nicorandil treatment at 20 weeks of age, when no release of cytochrome c from mitochondria had been observed although reduced cardiac function, cardiac hypertrophy, and cardiac fibrosis had already been observed in R120G TG mice. Although it is difficult to rule out other possibilities of protective actions such as focal vascular protection by nitric oxide and cellular protection via salcoremmal K(ATP) channel opening by nicorandil treatment, our findings imply that nicorandil treatment may be beneficial for the DRM via myocardial mitochondria protection and the inhibition of apoptotic cell death in the cardiomyocytes.

In our previous study, we found that R120G, which can cause DRM, led to increased perinuclear aggresome formations that contain amyloid oligomers, depressed cardiac function, and premature lethality [8]. Since it is hypothesized that amyloid oligomers can permeabilize cellular membranes and lipid bilayers, which may represent the primary toxic mechanism of amyloid pathogenesis [17], cellular toxicity induced by the amyloid oligomers is associated with mitochondrial function as well as induction of apoptotic cell death by cytochrome c release from mitochondria [8], [9]. In the present study, nicorandil treatment inhibited the release of cytochrome c from mitochondria, activation of caspase 3, and the occurrence of apoptotic cell death in the cardiomyocytes as well as *in-vivo* TG mouse hearts, while the levels of amyloid oligomers as well as of the aggregates were unchanged in both the cardiomyocytes and TG mice. These results indicate that nicorandil can display cardioprotective effects without modification of the mutant protein accumulation. Nicorandil has been reported to display cardioprotective characteristics including anti-apoptotic cell death behavior through activation of mitoK(ATP) channels [4]. Our results also showed that nicorandil as well as diazoxide, another mitoK(ATP) channel opener, decreased the cell toxicity induced by R120G, and 5-HD, a K(ATP) channel antagonist, blocked these protective effects by nicorandil and diazoxide in cardiomyocytes. Furthermore, nicorandil alone displayed cardioprotective effects in the R120G TG mice under relatively severe conditions, in which mitochondrial abnormalities such as cytochrome c leakiness occurred in the heart, while no beneficial effects were detected in this model under mild conditions. These results may imply that the opening of a K(ATP) channel in mitochondria can be cardioprotective against toxicity via mutant HSPB5, such as protein aggregates, and amyloid oligomers or can inhibit activation of the signaling pathway of apoptosis that is stimulated by the mutant protein accumulation in the cardiomyocytes. Thus, mitoK(ATP) channel opening may be beneficial for inhibition of disease progression via mitochondrial injury and apoptotic cell death in DRM, particularly when cardiac disease is severe.

The underlying mechanism(s) of protective action via opening the mitoK(ATP) channels through nicorandil treatment on cardiac disease in the DRM is unclear. It is known that the opening of mitoK(ATP) channels can be protective during ischemia-reperfusion. MitoK(ATP) channels were also proposed to be the end-effecter of IPC, a mechanism by which an exposure to brief periods of ischemia may provide protection against subsequent sustained ischemia/reperfusion injury [18], [19]. However, the mechanisms by which mitoK(ATP) channels exert their cardioprotective effects were also poorly understood. A mitoK(ATP) channel opener such as diazoxide led to a decrease in the inner membrane potential (ΔΨm) via modulation of Ca2+ influx and efflux through a ruthenium red-sensitive Ca2+ uniporter and a cyclosporin A-sensitive mitochondrial permeability transition pore, specific, voltage-dependent, nonselective high-conductance channels [20], [21]. Another study demonstrated that reduction in mitochondrial Ca2+ by diazoxide correlated with the post-ischemic recovery of the contractility in isolated, perfused hearts [22]. Furthermore, a significant increase in mitochondrial volume was observed by opening the mitoK(ATP) channel; this effect may protect mitochondria during ischemia-reperfusion by preserving the architecture of the intermembrane space with the consequent slowing of ATP hydrolysis [23]. It is suggested that the generation of reactive oxygen species leading to apoptotic cell death under ischemic/reperfused conditions is regulated by mitoK(ATP) channels in hearts [24], [25]. Because most of the previous findings related to the mitoK(ATP) channel were obtained using the isolated mitochondria, primary-cultured cardiomyocytes, and isolated, perfused hearts as acute effects, it is uncertain if the similar mechanism(s) of cardioprotection occur in chronic disease such as DRM by long-term treatment with a mitoK(ATP) channel opener. Since IONA study suggested that long-term treatment with nicorandil exerts cardioprtective effects via mitoK(ATP) channel opening, our findings concerning effects of nicorandil in DRM may be also exerted via mitoK(ATP) channel opening [1], [3]. Further study is needed to analyze the mechanism(s) of nicorandil in cardiac disease in the DRM.

Nicorandil treatment prevented the increase in BAX [26], an apoptotic inducible factor, and the decrease in BCL2, an anti-apoptosis factor [27], in cultured cardiomyocytes expressing mutant HSPB5 well as in the mutant TG mouse hearts. Apoptosis, a distinct type of cell death, is governed by a number of regulating genes from the BCL2 family, such as BCL2, BAX, BAK and BCL-X [28], [29]. The balance between expression of BCL2 and BAX plays an important role in the pathway of apoptotic and necrotic cell death [4], [28], [30]. These previous studies and our results suggest that the protein levels of apoptotic-related factors such as BCL2 and BAX may play a vital role in the initiation and development of cardiac disease in DRM. After administration of nicorandil, the protein level of BAX was attenuated while BCL2 levels were elevated in mitochondria, resulting in an elevated ratio of BCL2/BAX in comparison with those in untreated TG mice. Interestingly, a recent study showed that sustained BCL2 overexpression in R120G hearts prolonged these mice's survival [31]. This was associated with decreased mitochondrial abnormalities, restoration of cardiac function and attenuation of apoptosis [31]. The results of this study are quite similar to those in our present study, which indicates that nicorandil may prevent the pathogenesis and development of DRM via regulation of protein levels of the BCL family members such as BCL2 and BAX.

Although the protein levels of BCL2 and BAX were altered in the cardiomyocytes by the mutant HSPB5 and these alterations were blocked by nicorandil treatment *in vitro* and *in vivo*, these protein levels were independent of the mRNA expression in the hearts. Additionally, no correlation between the gene expression and the protein in BCL family members were observed in other studies [30], whereas direct correlation was seen in hypoxia-induced apoptosis in cardiomyocytes via nicorandil treatment [4]. The exact mechanism(s) causing the different results related to the BCL family between previous studies and the present study, and the divergent regulation between protein level and mRNA level in the BCL family in the hearts have not been fully clarified. It is known that BCL2 protein is cleaved by caspase, and a positive feedback between BCL2 and caspases is present [32]. Since it is possible that nicorandil contributes to the maintenance of the mitochondrial membrane potential by opening of mitoK(ATP) channels, mitochondrial membrane potential may play a role in turnover of BCL family members [28].

In summary, our results in the present study clearly indicate that treatment with nicorandil, the mitoK(ATP) channel opener, appeared to reduce mitochondrial impairment and apoptotic cell death and prolonged survival in the R120G TG mice. Nicorandil can inhibit the alteration of BCL2 and BAX in mitochondria in hearts from the R120G TG mice and can suppresses the mitochondrial death pathway through opening of the mitoK(ATP) channel, which may be involved in it's protective effect on DRM. Use of a mitoK(ATP) channel opener such as nicorandil may represent a new therapeutic strategy for patients with DRM.
